# Characterization and antimicrobial activity of lectins purified from three Egyptian leguminous seeds

**DOI:** 10.1186/s13568-020-01024-4

**Published:** 2020-05-15

**Authors:** Magda M. El-Araby, Einas H. El-Shatoury, Mervat M. Soliman, Hanan F. Shaaban

**Affiliations:** 1 Botany, Ain Shams University, Cairo, Egypt; 2 Microbiology, Ain Shams University, Cairo, Egypt

**Keywords:** Lectins, Antimicrobial, Scanning electron microscopy, Amino acid sequencing

## Abstract

Lectins are carbohydrate-binding proteins that play vital roles in many biological processes. In this study, lectins from three Egyptian cultivars (fava bean, lentil, and pea) were isolated by precipitation with different concentrations of ammonium sulfate. The purification process was performed by affinity chromatography using mannose agarose. The highest concentration of purified lectins (1.48 mg/g) was recorded in pea at 90% saturation. SDS-PAGE of the purified lectins revealed bands of low molecular weights (14 to 18 kDa). The complete amino acid sequences of purified lectins were assessed using mass spectrometry (MS), which indicated the presence of the peptides favin, p54, and psl in fava bean, lentil, and pea, respectively. The lectins showed antimicrobial activity. The highest inhibition zone (35 mm) was measured with lectin purified from lentil against *Staphylococcus aureus* ATCC 6538, followed by pea lectin (33.4 mm) against *Pseudomonas aeruginosa* ATCC 10145. To the best of our knowledge, the legume lectins in this study are the first lectins to exhibit antifungal activity against *Candida albicans*, with the maximum inhibition zone (25.1 mm) observed with purified lectins of fava bean. Additionally, the first scanning electron microscope (SEM) images showing agglutination and clumping of microbial cells exposed to tested lectins are provided. These findings proved that Egyptian legume lectins are distinct from other lectins reported in previous studies and demonstrated their potential as antimicrobial agents against human pathogenic microorganisms.

## Introduction

Lectins are proteins or glycoproteins that possess noncatalytic carbohydrate-binding sites (Van Damme et al. 2003). They differ from enzymes because their carbohydrate-binding properties never change, and they are unlike antibodies because they are not induced as an immune response (Faheina-Martins et al. 2012). Lectins have been reported in animals, plants, fungi, bacteria, and viruses, but the most well-known lectins are found in leguminous seeds (Lagarda-Diaz et al. 2017). They are responsible for innate immunity and defence mechanisms in plants (Van Damme et al. 1998).

Different types of legume lectins have been characterized, including fava bean (Hemperly et al. 1979), lentil seeds (Foriers et al. 1981), and pea seeds (Higgins et al. 1983). Lectins have sugar binding specificity, enabling them to recognize different glycotopes (Kamiya et al. 2012). Plant lectins are classified into groups, such as Gal/GalNAc-specific, glucose/mannose-specific, fucose-specific, GlcNAc-specific, and sialic acid-specific lectins (Goldstein and Portez 2012). The most biologically important proteins are mannose-binding lectins (Wong et al. 2008), which have been isolated and characterized from fava bean, lentil, and pea seeds (Dan et al. 2016).

Lectins cause cell agglutination, which is why lectins are called “agglutinins” **(**Sharon and Lis 2001). The ability of lectins to agglutinate human and other animal erythrocytes is considered the best method to detect lectin activity (Lam and Ng 2011). Lectins have many biological activities, such as antitumour (Lam and Ng 2009), antifungal and antibacterial activities (Klafke et al. 2013). The interaction of lectin with teichoic acids, teichuronic acids, peptidoglycans, and lipopolysaccharides present in the cell walls of bacteria is responsible for its antimicrobial activity (Ratanapo et al. 2001).

Lectins have attracted many biologists and scientists who are interested in the agriculture and medical fields (Movafagh et al. 2013). The emergence of infections and drug resistance make the existing antibiotics less effective. Currently, natural products, including lectins, are the best candidates for discovering new drugs (Patel et al. 2012).

In this work, lectins extracted from fava bean, lentil and pea were characterized by different methods. Moreover, the antimicrobial activity and photomicrographs were studied.

## Materials and methods

### Plant seeds and microbes

Three Egyptian cultivar seeds of *Vicia faba* (fava bean/Sakha 1), *Lens culinaris* (lentil/Giza 51), and *Pisum sativum* (pea/Master pea 38) were obtained from *the* Vegetable Breeding Research Department, Horticultural Research Institute, Agriculture Research Centre, Egypt. *Staphylococcus aureus* ATCC 6538, *Streptococcus mutants* ATCC 25175, *Klebsiella pneumonia, Escherichia coli* 0157: HZ ATCC 51659, and *Pseudomonas aeruginosa* ATCC 10145 were obtained from the Department of Microbiology, Faculty of Science, Ain Shams University. *Candida albicans* was purchased from the Regional Centre for Mycology and Biotechnology, Al-Azhar University, Egypt.

## Materials

Pro-Sieve Color Protein Markers (Cat number 50550, Lonza, USA). Dialysis of the tubing membrane with a cut off of 12 kDa (Spectra/Por, USA). Borosilicate glass column (C4169, Sigma Aldrich). d-Mannose-agarose (M6400-5ML, Sigma Aldrich). Human blood group types A, B, AB, and O were obtained from El-Araby Medical Centre Hospital, Cairo, Egypt.

### Lectin extraction

Extraction was carried out according to Zhang et al. (2008) with some modifications. Twenty-five grams of seeds were ground to powder in liquid nitrogen and homogenized in 250 ml of 50 mM phosphate buffer (pH 7.2) overnight at 4 °C. After filtration, the filtrate was centrifuged at 6000×*g* for 20 min, and the supernatant was fractionated by ammonium sulfate precipitation at 30%, 70%, and 90% saturation, sequentially. The pellet from each ammonium sulfate saturation was dissolved in 15 ml of phosphate buffer (pH 7.2) and dialysed overnight in the same buffer at 4 °C. The produced samples from the dialysis process were considered partially purified or dialysed lectins.

### Lectin purification

A total of 2 ml of mannose agarose was packed into a chromatography column and washed with phosphate buffer (pH 7.2). Then, 7 ml of sample was loaded onto the column and incubated for 30 min. Washing was carried out using 20 ml of equilibration buffer (50 mM Tris, pH 7.5, 100 mM NaCl, 5 mM CaCl_2_, 5 mM MgCl_2_), resulting in elution of unbound proteins that were monitored by reading UV absorption at 280 nm. Bound proteins were eluted using a linear gradient of elution buffer (50 mM Tris, pH 7.5, 100 mM NaCl, 5 mM CaCl_2_, 5 mM MgCl_2_, 500 mM mannose). Protein fractions were collected and dialysed overnight in phosphate buffer (pH 7.2) at 4 °C. The resulting proteins are called purified lectins.

### Protein concentration and separation

The protein concentration of lectin was determined according to the method of Bradford (1976) using bovine serum albumin (BSA) as a standard. SDS**-**PAGE using 12% separating gel and 5% stacking gel was performed according to Laemmli (1970).

### Haemagglutination activation test

The assay was performed in 96-well plates; two-fold serial dilutions of tested lectin samples (50 μl) in 5 mM phosphate buffer saline (pH 7.2) were added to 50 μl of 4% (w/v) RBCs. Then, the mixtures were incubated at 37 °C for one hour. A control (50 μl of PBS instead of protein solution and 50 μl of 4% cell suspension) was used as a reference (Wang et al. 2000). The haemagglutination activity was expressed as the titer (the reciprocal of the lowest concentration of protein at which visible agglutination of erythrocytes could be observed).

### Haemagglutination inhibition test

A serial two-fold dilution of sugars (galactose, glucose, maltose, mannose, and sucrose) was prepared in phosphate-buffered saline. All dilutions of tested sugars were mixed with an equal volume of 50 μl of lectin solutions (with positive haemagglutination activity) extracted from three leguminous seeds. The mixtures were incubated for one hour at room temperature and then mixed with 50 μl of a 4% human RBC suspension (Wang et al. 2000). The negative control contained 50 μl of protein solution and 50 μl of 4% RBCs. The inhibition of haemagglutination was expressed as positive or negative inhibition.

### Antibacterial and antifungal activity of lectin

Antimicrobial activity was tested using the agar-well diffusion method. Nutrient agar plates were inoculated with bacterial culture, while Sabouraud agar plates were inoculated with fungal cells (10^5^ CFU/ml for 24 h). Wells (8 mm) were filled with 100 μl of lectin solution extracted before and after using chromatography column, and 100 μl of 5 mM phosphate buffer solution pH 7.2 was used as a control. The plates were kept in a refrigerator overnight for diffusion. Then, the plates were incubated for 24 h at 37 °C. The average inhibition zone diameters (mm) around the well were measured (Ryan et al. 1996).

### Minimum inhibitory concentration

The minimum inhibitory concentration (MIC) was measured by the broth dilution test using an inoculum of 10^5^ CFU/ml for the tested microorganisms. Serial two-fold dilutions (with concentrations of 1000, 500, 250, 125, 62.5, 31.25, 15.63, 7.8, 3.9, 1.95, 0.98, 0.49 and 0.24 μg/ml) of the lectin fractions dialysed 90% for all tested seeds were prepared in 1 ml of brain heart infusion (BHI) medium. Then, 100 μl of the inoculum was added. Ampicillin, gentamycin, and amphotericin B were used as standard drugs for their gram-positive, gram-negative and antifungal activities, respectively. The minimum inhibitory concentration was defined as the lowest concentration of the tested samples that inhibited the visible growth of bacteria after 24 h and fungi after 48 h (Rex et al. 2001).

### Scanning electron microscopy examination

The samples were fixed using 25% glutaraldehyde and dehydrated by serial dilution of ethanol using an automatic tissue processor (Leica EMTP). Samples were dried using a CO_2_ critical point drier (Tousimis Autosamdri-815). They were coated by a gold sputter coater (SPI-Module). Finally, the samples were examined by scanning electron microscopy (JEOL-JSM-5500 LV) using high vacuum mode at the Regional Centre of Mycology and Biotechnology, Al-Azhar University, Cairo, Egypt.

### Amino acid sequence analysis

The gel bands of fava bean, lentil, and pea lectins (18, 14, and 17 kDa, respectively) were subjected to cysteine reduction and carbamidomethylation followed by in-gel trypsin digestion. The extracted peptides were analysed by an Agilent UHD 6530 Q-Tof mass spectrometer at the Mass Spectrometry Facility of the Advanced Analysis Centre, University of Guelph, Canada. The raw data files were loaded directly into PEAKS 85 software (Bioinformatics Solutions Inc), where the data were refined and subjected to de Novo sequencing and database searching.

### Statistical analysis

Statistical analysis was performed by one-way ANOVA (Armstrong et al. 2000).

## Results

### Determination of lectin concentration

**Fig. 1:**
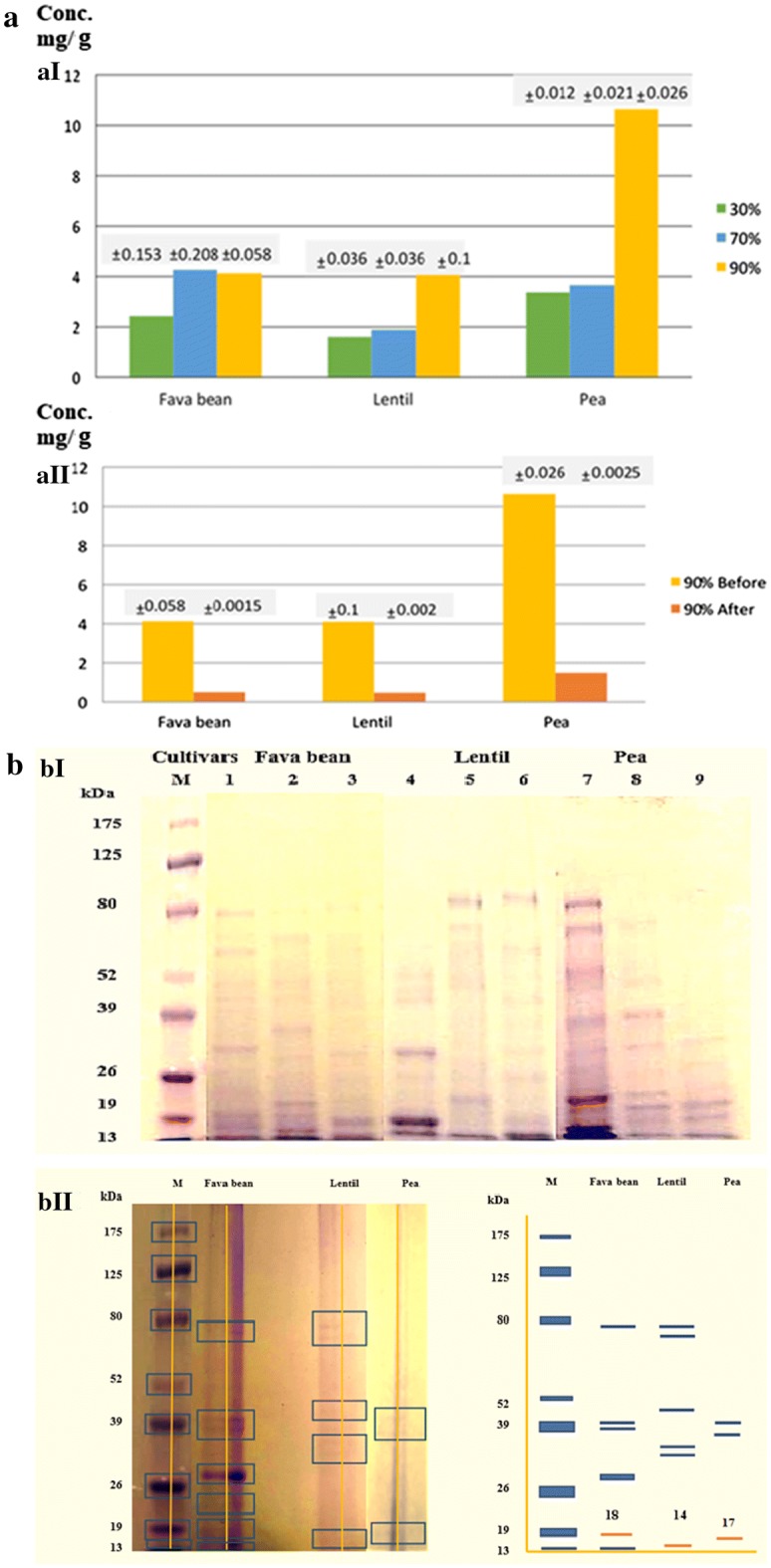
**a-aI** Concentrations (mg/g) of fractionated lectins extracted from three leguminous seeds (fava bean, lentil, and pea). Lectin fractionation was obtained by precipitation with ammonium sulfate at 30, 70 and 90% saturations followed by extensive dialysis. Results are expressed as mean of three replicates with ± standard deviation. **a-aII** Concentrations (mg/g) of 90% fraction of lectins extracted from (fava bean, lentil, and pea) before and after purification by affinity chromatography. **b-bI** SDS-PAGE profiles of fractionated partially purified lectins extracted from three leguminous seeds after Coomassie blue staining. Lanes 1, 4, 7 represent 90% saturation. Lanes 2, 5, 8 represent 70% saturation. Lanes 3, 6, 9 represent 30% saturation. **b-bII** SDS-PAGE profiles and its electropherogram of purified lectins obtained by affinity chromatography after Coomassie blue staining. The purified lectins subunits bands appear at molecular weight 18, 14, 19 kDa of fava bean, lentil, and pea, respectively

The crude extracts of three leguminous seeds were fractionally precipitated with ammonium sulfate (saturation 30%, 70%, and 90%) followed by overnight dialysis. The largest yield of the dialysed lectins was reported at 90% saturation for the tested seeds, and the highest concentration (10.64 mg/g) of dialysed lectins was recorded in pea (Fig. 1a-aI). The 90% saturation fractions of fava bean, lentil, and pea were purified by affinity chromatography using mannose agarose. The yield of purified lectins was determined and is shown in Fig. 1a-aII.

Lectin fractions (30%, 70%, and 90%) of three legume seeds were loaded on SDS-PAGE gels (Fig. 1b-bI). After affinity chromatography, the 90% saturation fractions of purified lectins were loaded on SDS-PAGE, and the electropherogram showed the patterns of the purified lectins (Fig. 1b-bII).

### Haemagglutination activation and inhibition tests

The dialysed lectin and its purified form (90% fraction) agglutinated all types of human blood groups. The total haemagglutination activity, specific haemagglutination activity, and fold purification of lectins were recorded (Table 1).

**Table 1:** Hemagglutination activity of partially and purified lectins (90% fractions) extracted from fava bean, lentil, and pea on human blood groups (A, B, AB, and O)

Seeds	Purity of lectins	Conc. protein (mg/g)	Total protein (mg)	Total hemagglutination activity (titer)				Specific hemagglutination activity (titer/mg)				Fold of purification			
				A	B	AB	O	A	B	AB	O	A	B	AB	O
Fava bean	Partially purified	4.13	103.25	16	32	128	32	0.15	0.30	1.24	0.30	1	1	1	1
	Purified	0.50	12.5	8	16	64	32	0.67	1.28	5.12	2.56	4.46	4.27	4.12	8.5
Lentil	Partially purified	4.1	102.5	32	32	64	64	0.31	0.31	0.62	0.62	1	1	1	1
	Purified	0.48	12	8	16	16	16	0.67	1.33	1.33	1.33	2.16	4.29	2.15	2.15
Pea	Partially purified	10.64	266	8	64	64	128	0.03	0.24	0.24	0.48	1	1	1	1
	Purified	1.48	37	8	32	32	64	0.22	0.86	0.86	1.73	7.33	3.58	3.58	3.60

Titer, the reciprocal of the heights dilution of protein solution showing visible hemagglutination

The haemagglutination inhibition test was performed in the presence of different sugars, and the results in Table 2 indicated that the haemagglutination inhibition of lectins and their purified forms of fava bean, lentil, and pea was similar; the lectins were inhibited by glucose and mannose sugars at different degrees but not inhibited by galactose, maltose, and sucrose.

**Table 2:** Hemagglutination inhibition of partially purified lectins (70%, and 90% fractions) and purified lectins (90% fractions) extracted from fava bean, lentil, and pea using different sugars

Seeds	Purity of Lectin	Fractions (%)	Hemagglutination inhibition				
			Sugars				
			Galactose	Glucose	Maltose	Mannose	Sucrose
Fava bean	Partially purified	70	−	++	−	++	−
		90	−	+++	−	+++	−
	Purified	90	−	+++	−	+++	−
Lentil	Partially purified	70	−	+	−	++	−
		90	−	+++	−	+++	−
	Purified	90	−	+++	−	+++	−
Pea	Partially purified	70	−	++	−	++	−
		90	−	+++	−	+++	−
	Purified	90	−	+++	−	+++	−

The number of + signs indicate the amplitude of relative hemagglutination inhibition

+ = positive hemagglutination inhibition; − = negative hemagglutination inhibition

### Antimicrobial activity

The partially purified lectins of three tested seeds at 30%, 70%, and 90% saturation showed antifungal activity against *Candida albicans* (Table 3). Furthermore, the lectins purified by affinity chromatography inhibited the growth of *Candida albicans* (Table 4).

**Table 3:** Antimicrobial activity of partially purified lectins (30, 70, and 90% fractions) extracted from fava bean, lentil, and pea against tested microorganisms

Seeds	Lectin fractions (%)	Inhibition zone diameters of the tested microorganisms (mm)					
		*Escherichia coli* (−)	*Klebsiella pneumonia* (−)	*Pseudomonas aeruginosa* (−)	*Staphylococcus aureus* (+)	*Streptococcus mutants* (+)	*Candida albicans*
Fava bean	30	NI	4.57 ± 0.06	5.27 ± 0.06	8.13 ± 0.23	5.43 ± 0.21	13.10 ± 0.10
	70	NI	10.13 ± 0.15	15.07 ± 0.12	12.03 ± 0.06	11.43 ± 0.40	12.27 ± 0.25
	90	NI	14.47 ± 0.23	20.43 ± 0.15	16.27 ± 0.31	14.40 ± 0.36	17.40 ± 0.17
Lentil	30	NI	6.23 ± 0.25	4.40 ± 0.10	4.33 ± 0.25	4.47 ± 0.25	9.47 ± 0.15
	70	NI	11.60 ± 0.10	8.57 ± 0.06	14.37 ± 0.21	13.13 ± 0.12	12.23 ± 0.25
	90	NI	16.07 ± 0.12	16.23 ± 0.25	24.37 ± 0.21	18.33 ± 0.15	15.30 ± 0.10
Pea	30	NI	15.13 ± 0.15	4.17 ± 0.15	3.53 ± 0.25	2.30 ± 0.10	6.30 ± 0.20
	70	NI	16.17 ± 0.15	17.30 ± 0.30	5.37 ± 0.21	3.70 ± 0.20	8.43 ± 0.15
	90	NI	25.07 ± 0.06	23.30 ± 0.20	18.50 ± 0.27	12.27 ± 0.23	14.50 ± 0.20
P-value	P 0.05	N.S.	0.0001	0.0001	0.0001	0.0001	0.0001
			Sig.	Sig.	Sig	Sig	Sig
	P 0.01	N.S.	0.0001	0.0001	0.0001	0.0001	0.0001
			Sig.	Sig.	Sig	Sig	Sig

Antimicrobial activity measured as inhibition zones diameters (mm)

The results are represented as mean of three replicates with ± standard deviation

*NI* no inhibition zone, *Sig.* significant, *N.S.* not significant, (−) gram-negative bacteria, (+) gram-positive bacteria

P-value ≤ 0.05 and 0.01 considered statistically significant (95 and 99% confidence interval)

**Table 4:** Antimicrobial activity of purified lectins (fraction 90%) extracted from fava bean, lentil, and pea against tested microorganisms

Seeds	Lectin fractions (90%)	Inhibition zone diameters of the tested microorganisms (mm)					
		*Escherichia coli* (−)	*Klebsiella pneumonia* (−)	*Pseudomonas aeruginosa* (−)	*Staphylococcus aureus* (+)	*Streptococcus mutants* (+)	*Candida albicans*
Fava bean		NI	23.33 ± 0.58	24.00 ± 1.00	21.40 ± 0.10	22.17 ± 0.15	25.13 ± 0.15
Lentil		NI	14.00 ± 1.00	20.13 ± 0.15	35.100 ± 0.10	26.33 ± 0.21	23.13 ± 0.23
Pea		NI	29.23 ± 0.25	33.40 ± 0.17	27.300 ± 0.20	20.33 ± 0.31	19.17 ± 0.15
P-value							
P 0.05		N.S.	0.0001	0.0001	0.0001	0.0001	0.0001
			Sig.	Sig.	Sig	Sig	Sig
P 0.01		N.S.	0.0001	0.0001	0.0001	0.0001	0.0001
			Sig.	Sig.	Sig	Sig	Sig

Antimicrobial activity measured as inhibition zones diameters (mm)

The results are represented as mean of three replicates with ± standard deviation

*NI* no inhibition zone; mm millimeter, *Sig.* significant, *N.S.* not significant, (−) gram-negative bacteria, (+) gram-positive bacteria

P-value ≤ 0.05 and 0.01 considered statistically significant (95 and 99% confidence interval)

On the other hand, the partially purified lectins of the three tested seeds at 30%, 70%, and 90% saturation displayed antibacterial activity against *Staphylococcus aureus, Streptococcus mutants*, *Pseudomonas aeruginosa*, and *Klebsiella pneumonia* but not *Escherichia coli* (Table 3). Moreover, the purified lectins obtained by affinity chromatography showed a similar pattern of inhibition (Table 4).

### Minimum inhibitory concentration (MIC)

Table 5 shows the MIC values of lectins (90% fractions) against the tested bacteria and fungi. The inhibition ranged from 1.95 to 250 µg/ml. The results showed that lentil lectins had the lowest minimum inhibitory concentration (1.95 µg/ml) against *Staphylococcus aureus.*

**Table 5:** Minimum inhibitory concentration (MIC) using series of two-fold dilutions of lectins (fraction 90%) of three leguminous seeds against the tested microorganisms

Tested microorganisms	Minimum inhibitory concentration (µg/ml)			
	Samples of dialysed Lectins			
	Fava bean	Lentil	Pea	Standards
*Escherichia coli*	NI	NI	NI	*Gentamicin*
				NI
*Klebsiella pneumonia*	15.63	3.9	62.5	0.98
*Pseudomonas aeruginosa*	62.5	15.63	125	3.9
*Staphylococcus aureus*	15.63	1.95	125	*Ampicillin*
				0.98
*Candida albicans*	31.25	3.9	250	*Amphotericin B*
				1.95

*NI* no inhibition

### Scanning electron microscope examination (SEM)

**Fig. 2:**
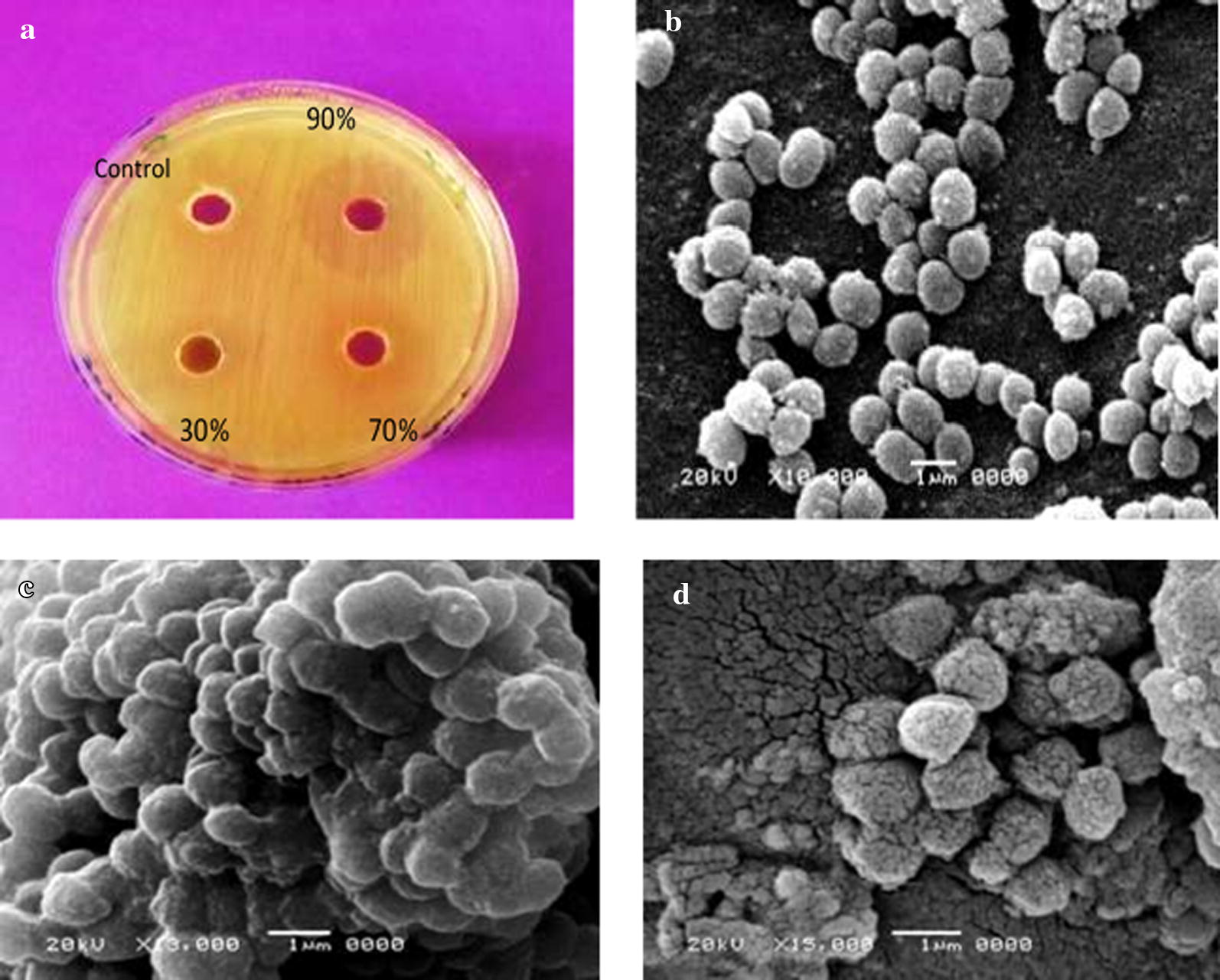
**a** Shows the effect of 100 µl of lectin (30,70, 90% fractions) isolated from fava bean seeds against Gram-positive bacteria *Staphylococcus aureus*. Scanning electron microscope images. **b** Represent bacteria *Staphylococcus aureus* without treatment of lectins (**c**) and (**d**) with treatment by 100 µl (90% fraction) dialysed lectin and its purified form respectively

**Fig. 3:**
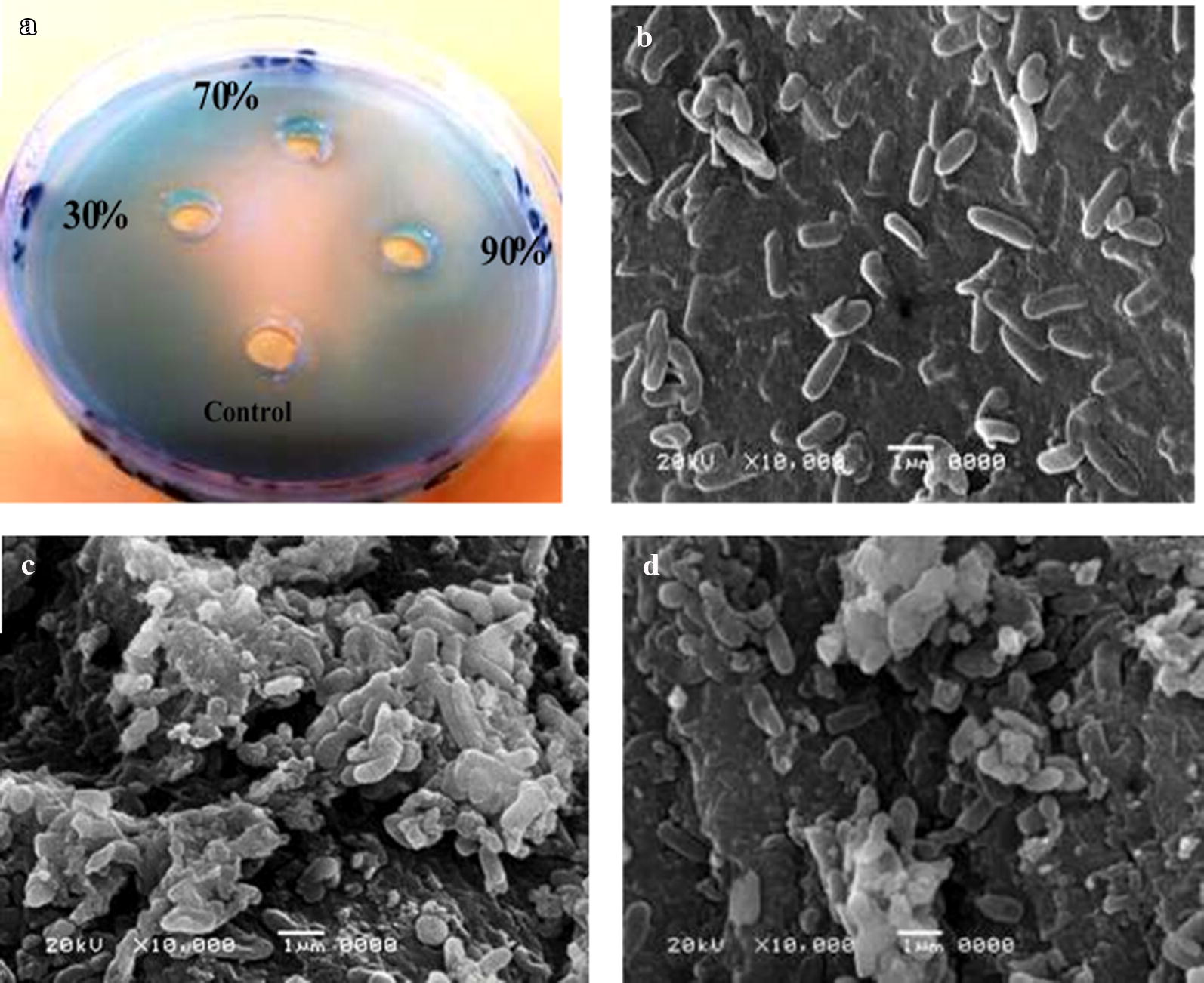
**a** Shows the effect of 100 µl lectin of lentil seeds (30,70, 90% fraction) against Gram-negative bacteria *Pseudomonas aeruginosa*. Scanning electron microscope images. **b** Represent bacteria *Pseudomonas aeruginosa* without treatment of lectins (**c**) and (**d**) with treatment by 100 µl (90% fraction) dialysed lectin and its purified form respectively

**Fig. 4:**
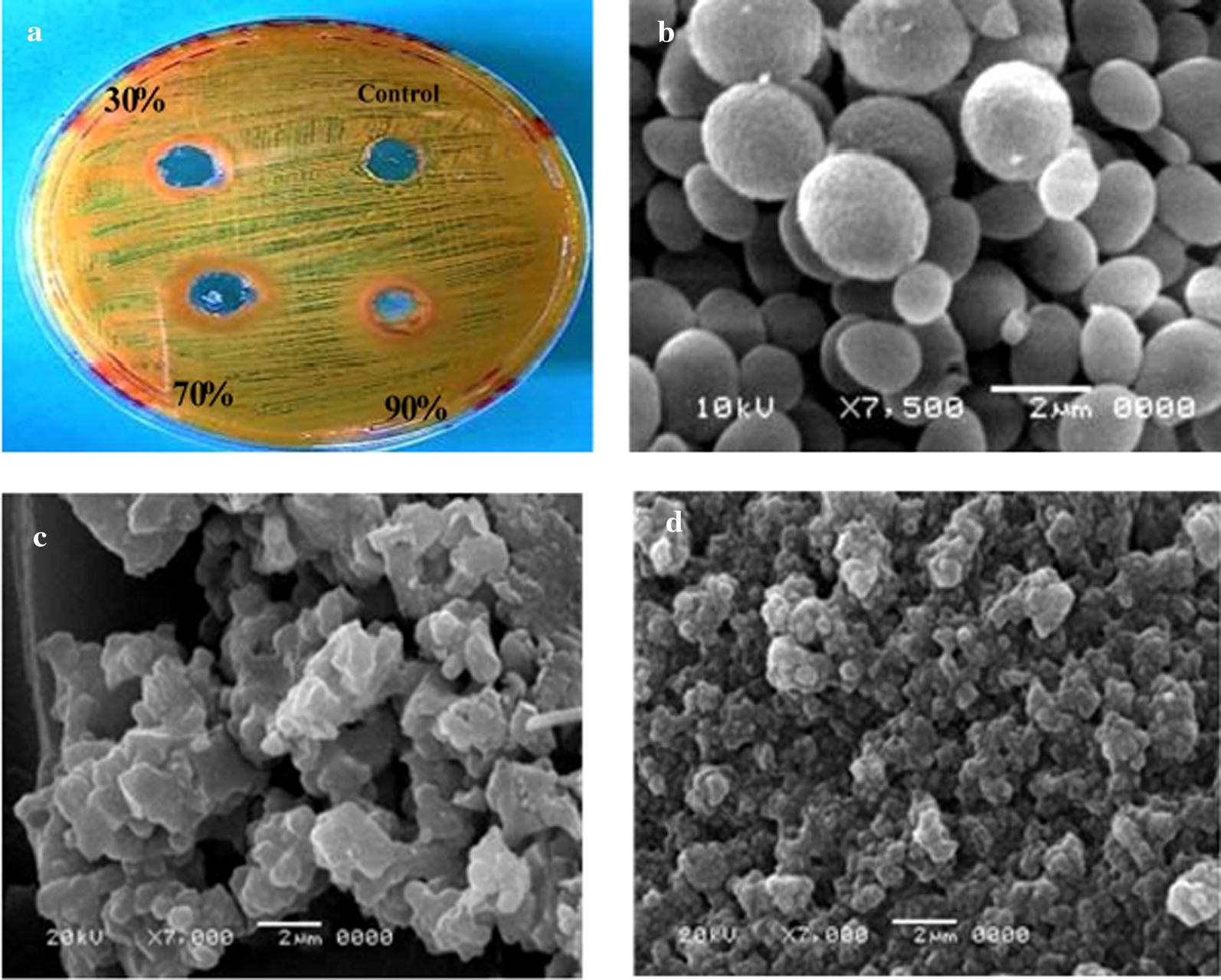
**a** Shows the effect of 100 µl lectin of pea seeds (30, 70, 90% fraction) against tested fungi *Candida albicans*. Scanning electron microscope images. **b** Represent fungi *Candida albicans* without treatment of lectins (**c**) and (**d**), with treatment by 100 µl (90% fraction) dialysed lectin and its purified form respectively

The photomicrographs of tested microorganisms before and after treatment by partially purified lectins and purified lectins indicated that lectins had an effect against the tested microbial cells. Figures 2, 3 and 4 show the activity of partially purified lectins and their purified forms (90% fraction) of fava bean, lentil, and pea, against *Staphylococcus aureus, Pseudomonas aeruginosa*, and *Candida albicans* before and after treatment, respectively. The results proved that lectins are able to mediate strong agglutination and destruction effects on microbial cells.

### Amino acid sequences of fava bean, lentil, and pea lectins

**Fig. 5:**
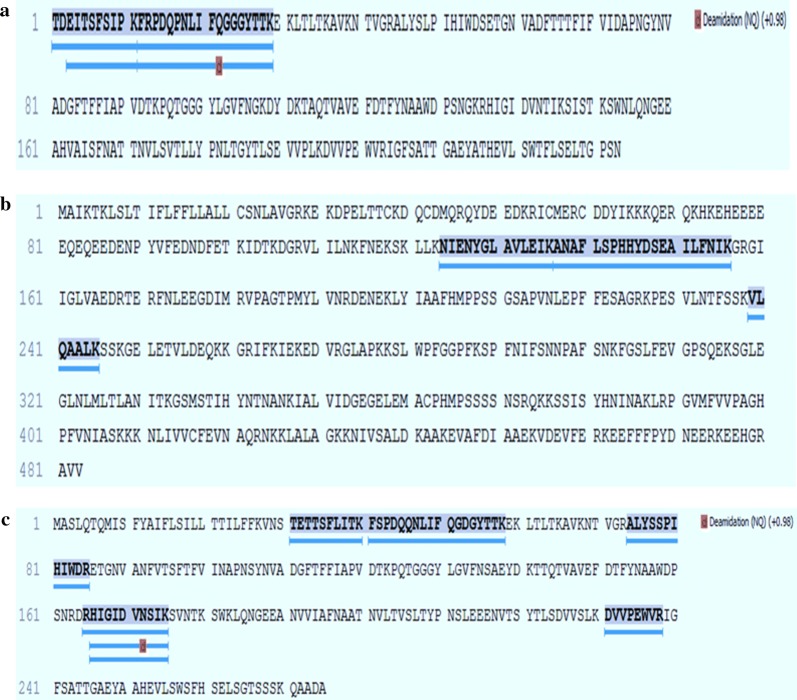
**a** Shows amino acid sequences fava bean lectins (Favin (P02871|LEC_VICFA). However, data is often searched the variable modification deamidation NQ (+0.98 Da) of asparagines (N) and glutamines (Q). **b** Shows amino acid sequences lentil lectins. **c** Shows amino acid sequences pea (psl) lectins. However, data is often searched the variable modification deamidation NQ (+ 0.98 Da) of asparagines (N) and glutamines (Q)

The purified lectin bands of fava bean, lentil, and pea with molecular weights of 18, 14, and 17 kDa, respectively, were subjected to amino acid sequence analysis. Tables 6, 7 and 8 show the complete amino acid sequences of the tested purified lectins. Moreover, Fig. 5 indicates the presence of the peptides favin, p54, and psl in fava bean, lentil, and pea samples, respectively.

**Table 6:** Showed all data (PEAKS 8.5 software) of the complete amino acid sequences of fava bean lectin (Favin P02871|LEC_VICFA)

Peptide sequences (this study)	− 10lgP	Mass	Length	ppm	m/z	RT	Scan	Intensity sample	Peptide location		PTM
									Start	End	
TDEITSFSIPK.F	45.59	1236.62	11	3.2	619.32	18.45	2968	3.28E4	1	11	
K.FRPDQPNLIFQGGGYTTK.E	39.35	2038.03	18	3.5	680.35	18.38	2956	4.28E4	12	29	
K.FRPDQPNLIFQ (+ 0.98)GGGYTTK.E	30.45	2039.01	18	5.0	680.68	18.70	3011	1.06E4	12	29	Deamidation (NQ)
D.EITSFSIPK.F	21.23	1020.55	9	− 3.8	511.28	17.77	2852	1.32E3	3	11	
Total 4 peptides											

Peptide sequence (previous study)	Peptide location (β chain)	References
TDEITSFSIPK	122–132	Hemperly et al. (1979)
FRPDQPNLI	133–141	
EITSFSIPK	124–132	

− 10lgP = Conversion form of P-value in PEAKS 8.5 software where P-value of 1% is equivalent to − 10lgP of 20

*ppm* delta mass is the deviation of measured mass from the theoretical mass of the peptide, in ppm, *m/z* mass of ions (in gas phase) per charge, *RT* relatively accurate prediction of peptide, *PTM* post translational modification, *NQ* = variable modification deamidation NQ (+ 0.98 Da) of asparagines (N) and glutamines (Q)

**Table 7:** Showed all data (PEAKS 8.5 software) of the complete amino acid sequences of lentil lectin

Peptide sequences (this study)	− 10lgP	Mass	Length	ppm	m/z	RT	Scan	Intensity sample	Peptide location		PTM
									Start	End	
K.NIENYGLAVLEIK.A	43.18	1474.80	13	5.8	738.41	22.35	4388	7.66E3	124	136	
K.ANAFLSPHHYDSEAILFNIK.G	26.03	2286.14	20	− 9.1	572.54	20.48	3985	0	137	156	
K.VLQAALK.S	23.74	741.47	7	9.7	371.75	12.84	2366	1.28E4	239	245	
Total 3 peptides											

These peptide sequences were matched and compared using NCBI BLAST. The result of purified lentil lectin, in this study, showed high similarity (100%) to p54 protein that was found in pea

− 10lgP = Conversion form of P-value in PEAKS 8.5 software where P-value of 1% is equivalent to -10lgP of 20

*ppm* delta Mass is the deviation of measured mass from the theoretical mass of the peptide, in ppm, *m/z* mass of ions (in gas phase) per charge, *RT* relatively accurate prediction of peptide, *PTM* post translational modification

**Table 8:** Showed all data (PEAKS 8.5 software) of the complete amino acid sequences of pea lectin (psl)

Peptide Sequences (This study)	− 10lgP	Mass	Length	ppm	m/z	RT	Scan	Intensity sample	Peptide location		PTM
									Start	End	
R.HIGIDVNSIK.S	62.31	1094.61	10	12.0	365.88	15.58	2621	6.72E5	166	175	
S.TETTSFLITK.F	40.05	1139.61	10	1.7	570.81	16.93	2848	6.15E3	31	40	
R.ALYSSPIHIWDR.E	38.29	1456.75	12	7.1	486.59	19.30	3256	7.71E3	74	85	
R.HIGIDVN (+ 0.98) SIK.S	38.07	1095.59	10	10.9	366.21	15.19	2553	1.31E4	166	175	Deamidation (NQ)
K.FSPDQQNLIFQGDGYTTK.E	34.19	2057.97	18	2.4	686.99	18.74	3159	0	41	58	
D.RHIGIDVNSIK.S	32.44	1250.71	11	4.1	417.91	14.28	2399	1.55E5	165	175	
K.DVVPEWVR.I	30.86	998.52	8	7.2	500.27	18.34	3090	1.13E4	231	238	
Total 7 peptides											

Peptide sequence of (previous study)	Peptide location (β chain)	References
HIGIDVNSIK	136–145	Sharon and Lis (1990)
TETTSFLITK	1–9	
ALYSSPIHIWDR	42–53	
FSPDQQNLIFQGDGYTTK	10–27	
PEWVR	200–204	
DVVPEWVR	83–90	Hemperly et al. (1979)

− 10lgP = Conversion form of P-value in PEAKS 8.5 software where P-value of 1% is equivalent to − 10lgP of 20

*ppm* delta Mass is the deviation of measured mass from the theoretical mass of the peptide, in ppm, *m/z* mass of ions (in gas phase) per charge, *RT* relatively accurate prediction of peptide, *PTM* post translational modification, *NQ* variable modification deamidation NQ (+ 0.98 Da) of asparagines (N) and glutamines (Q)

## Discussion

Lectins have become vital tools and play important roles in different biological activities, especially in biochemistry, molecular biology, immunology, pharmacology, medicine, and clinical analysis (Silva et al. 2016). Leguminous seeds are the best group of lectins, as they represent a rich source of lectins (Sun and Yang 2011). Furthermore, up to 5% of the total protein of seeds is lectin (Peumans and Van Damme 1995).

In the present study, after the purification process, the yield of pea lectins was 1.48 mg/g, which was nearly the same value (1.6 mg/g) that was obtained by Sitohy et al. (2007). The amounts of lectins purified from fava beans and lentils in this study were 0.481 and 0.503 mg/g, respectively, whereas Zhang et al. (2008) **o**btained fava beans and lentil lectin concentrations of 0.3 and 0.6 mg/g, respectively.

Lectins are classified into two groups according to their mannose/glucose binding specificity: one of these groups had four identical subunits, and the other had two light chain α subunits and two heavy chain β subunits similar to the lectins obtained from fava bean, lentil and pea (Goldstein and Portez 2012).

In this study, the molecular weight of favin lectins (18 kDa) was close to that of the β subunit (18.7) of favin lectin obtained by Hemperly et al. (1979). Moreover, the purified lentil lectins gave a molecular weight of 14 kDa, which is close to the molecular weight of the lentil lectin subunit (17 kDa) (Chan et al. 2015) and differs from the molecular weight of the polypeptide chains of lentil lectins (24.5 kDa) obtained by Howard et al. (1971). Our results showed that the purified pea lectin gave a band at a molecular weight of 17 kDa. This result agreed with the molecular weight of β subunits of pea lectins obtained by Trowbridge (1974) and differs from the molecular weight of β subunits of pea lectins (19.3 kDa) reported by Sitohy et al. (2007).

The agglutination of blood groups and the detection of lectins are based on the presence or absence of specific glycoproteins on red blood cells that lectins can bind (Moreira et al. 1991). The present study observed that partially purified lectins and their purified forms agglutinated all types of human red blood cells, indicating that lectins have a nonspecific blood group nature.

Verification of haemagglutination activity was carried out by an inhibition test using different sugars (Tsivion and Sharon 1981) since lectins had the ability to bind with one sugar (Sultan et al. 2009) or two sugars (Devi et al. 2008). The results of this study showed that lectins extracted from fava bean, lentil, and pea were inhibited by two sugars, namely, glucose and mannose.

Plant lectins with antibacterial and antifungal activities are natural bioactive molecules that control pathogens causing diseases in humans (Jandú et al. 2017; Breitenbach et al. 2018).

Many studies have detected that lectins with different carbohydrate specificities are able to recognize most components of the bacterial cell wall, such as muramic acid, N-acetylglucosamine, N-acetylmuramic acid and muramyl dipeptide (Ayouba et al. 1994). In this study, the dialysed lectins and their purified forms extracted from seeds had antibacterial activity against *Pseudomonas aeruginosa* and *Staphylococcus aureus*. These results agreed with the results of Nair et al. (2013).

To the best of our knowledge, this study is the first to report the antibacterial effect of partially purified and purified lectins extracted from tested seeds against *Klebsiella pneumonia* and *Streptococcus mutants* ATCC 25175 using the agar well diffusion method.

Chitin is the key component of the cell wall of fungi. Some lectins are chitin-binding proteins (Fakhoury and Woloshuk 2001), and other lectins are carbohydrate-binding proteins (Klafke et al. 2013). These properties suggested the antifungal effect of lectins. In this study, the observed antifungal activity of the tested legume lectins against *Candida albicans* agreed with the results obtained from another study on legume lectins from *Archidendron jiringa* seeds (Charungchitrak et al. 2011).

In this work, the lowest minimum inhibitory concentrations (MICs, μg/ml) of the tested leguminous lectins were enough to exhibit antibacterial effects against *Klebsiella pneumonia*, *Pseudomonas aeruginosa*, and *Staphylococcus aureus*. These results matched with the MICs of other lectins obtained from *Eugenia uniflora* seeds Oliveira et al. (2008).

To the best of our knowledge, this is the first report of tested legume lectins (fava bean, lentil, and pea) that inhibit the growth of *Candida albicans* using the agar-well diffusion method. Moreover, the minimum inhibitory concentration (MIC) of the purified lectins (90% fractions) against *Candida albicans* was recorded. Further investigation is required to find and optimize the mechanism of inhibition.

The results of this study, to our knowledge, are the first report of photomicrographs of scanning electron microscopy (SEM) showing the clumping and agglutination of *Staphylococcus aureus*, *Pseudomonas aeruginosa*, and *Candida albicans* after treatment with dialysed and purified lectins of fava bean, lentil, and pea, respectively.

A previous study reported that favin lectin is composed of two polypeptide chains, the α chain and β chain, and the β chain of favin lectins (18.7 kDa) contains approximately 180 amino acid residues. Furthermore, the favin lectin contains a small amount of a third β chain (Hemperly et al. 1979; Hopp et al. 1982).

In this study, the amino acid sequence analysis revealed that purified favin lectins contain 161 residues of 4 peptides that consist of 56 residues. The complete amino acid sequence of the peptide β chain of favin, reported here, is similar to the sequences reported earlier (TDEITSFSIPK, FRPDQPNLI, and EITSFSIPK), but the amino acids differ in their positions due to the different isolation procedures resulting in a different organization of the peptide chains (Hemperly et al. 1979; Sharon and Lis 1990).

Different cultivars of lentil seeds can generate lectins with variable activities (Chan et al. 2015). In previous studies, the α chain of lentil lectin was shown to be homologous to the α chain of pea lectin (Foriers et al. 1981). The pea lectin sequence showed a high degree of homology with the amino acid sequences of lentil lectins (Higgins et al. 1983). Moreover, the protein composition of lentil seeds was similar to that found in other species, such as pea seeds (Gallardo et al. 2001; Bourgeois et al. 2009).

The previous results agreed with our study in which the complete amino acids of lentil lectins revealed p54 proteins that are found in pea. The amino acid sequence of lentil lectin in this study contained 481 amino acid residues from 3 peptides. The sequences of the resulting peptides were found to be NIENYGLAVLEIK, ANAFLSPHHYDSEAILFNIK, and VLQAALK. A protein BLAST search of these sequences revealed that they matched the p54 protein (100% identical) that was found in pea. Further studies are required to investigate the homology between amino acid sequences of leguminous lectins that may lead to an understanding of their role in many biological activities.

A previous study of the amino acid sequences of β subunits (I7 kDa) of pea lectins reported that lectins contain the following peptide sequences: HIGIDVNSIK, TETTSFLITK, ALYSSPIHIWDR, FSPDQQNLIFQGDGYTTK, PEWVRDVV, and PEWVR (Hemperly et al. 1979; Sharon and Lis 1990). These results agreed with the amino acid sequences of pea lectins (psl) reported in our results.

In conclusion, extraction, purification, and molecular characterization are great approaches to study lectins. In this study, the purified lectins from fava bean, lentil, and pea were characterized by different methods, including mass spectrometry (MS) sequence analysis. In addition, we demonstrated their ability to inhibit the growth of gram-positive bacteria such as *Staphylococcus aureus* ATCC 6538 and *Streptococcus mutants* ATCC 25175 and gram-negative bacteria such as *Pseudomonas aeruginosa* ATCC 10145 and *Klebsiella pneumonia.* Lectins also showed antifungal activity against *Candida albicans.* The inhibition of microbial growth might be attributed to the clumping and agglutination effect of lectins on microbial cells, as observed by scanning electron microscopy (SEM). The antimicrobial activity of legume lectins must be considered beneficial. The similarity between the amino acid sequences of lentil and pea lectins needs further investigation.

## Data Availability

The corresponding author is responsible for providing all experimental data upon request.

## References

[CR1] Armstrong RA, Slade SV, Eperjesi F (2000). An introduction to analysis of variance (ANOVA) with special reference to data from clinical experiments in optometry. Ophthalmic Physiol Opt.

[CR2] Ayouba A, Causse H, Van Damme EJ, Peumans WJ, Bourne Y, Cambillau C, Rouge P (1994). Interactions of plant lectins with the components of the bacterial cell wall peptidoglycan. Biochem Syst Ecol.

[CR3] Bourgeois M, Jacquin F, Savois V, Sommerer N, Labas V, Henry C, Burstin J (2009). Dissecting the proteome of pea mature seeds reveals the phenotypic plasticity of seed protein composition. Proteomics.

[CR4] Bradford MM (1976). A rapid and sensitive method for the quantitation of microgram quantities of protein utilizing the principle of protein-dye binding. Anal Biochem.

[CR5] Breitenbach BC, Marcelino SS, Felix OW, de Moura MC, Viana PE, Soares GF, dos Santos CM (2018). Lectins as antimicrobial agents. J Appl Microbiol.

[CR6] Chan YS, Yu H, Xia L, Ng TB (2015). Lectin from green speckled lentil seeds (*Lens culinaris*) triggered apoptosis in nasopharyngeal carcinoma cell lines. Chin Med.

[CR7] Charungchitrak S, Petsom A, Sangvanich P, Karnchanatat A (2011). Antifungal and antibacterial activities of Lectin from the seeds of *Archidendron jiringa Nielsen*. Food Chem.

[CR8] Dan X, Liu W, Ng TB (2016). Development and applications of lectins as biological tools in biomedical research. Med Res Rev.

[CR9] Devi SK, Devi LI, Singh LR (2008). Purification and characterization of a new dimeric mannose/glucose-binding iso Lectin from *Vicia tetrasperma* L. Schreber. Prep Biochem Biotechnol.

[CR10] Faheina-Martins GV, da Silveira AL, Cavalcanti BC, Ramos MV, Moraes MO, Pessoa C, Araújo DA (2012). Antiproliferative effects of lectins from *Canavalia ensiformis* and *Canavalia brasiliensis* in human leukemia cell lines. Toxicol In Vitro.

[CR11] Fakhoury AM, Woloshuk CP (2001). Inhibition of growth of *Aspergillus flavus* and fungal α-amylases by a lectin-like protein from *Lablab purpureus*. Mol Plant Microbiol Int.

[CR12] Foriers A, Lebrun E, Van Rappenbusch R, DeNeve R, Strosberg AD (1981). The structure of the lentil (*Lens culinaris*) lectin amino acid sequence determination and prediction of the secondary structure. J Biol Chem.

[CR13] Gallardo K, Job C, Groot SP, Puype M, Demol H, Vandekerckhove J, Job D (2001). Proteomic analysis of *Arabidopsis* seed germination and priming. Plant Physiol.

[CR14] Goldstein I, Portez RD, Gabius HJ, Gabius S (2012). Isolation, physicochemical characterization, and carbohydrate binding specificity of lectins. The lectins properties, functions, and applications in biology and medicine.

[CR15] Hemperly J, Hopp TP, Becker JW, Cunningham BA (1979). The chemical characterization of favin, a lectin isolated from *Vicia faba*. J Biol Chem.

[CR16] Higgins TJ, Chandler PM, Zurawski G, Button SC, Spencer D (1983). The biosynthesis and primary structure of pea seed lectin. J Biol Chem.

[CR17] Hopp TP, Hemperly JJ, Cunningham BA (1982). Amino acid sequence and variant forms of favin, a lectin from *Vicia faba*. J Biol Chem.

[CR18] Howard IK, Sage HJ, Stein MD, Young NM, Leon MA, Dyckes DF (1971). Studies on a phytohemagglutinin from the lentil II. Multiple forms of *Lens culinaris* hemagglutinin. J Biol Chem.

[CR19] Jandú JJ, Moraes NR, Zagmignan A, de Sousa EM, Brelaz-de-Castro MC, dos Santos CM, da Silva LC (2017). Targeting the immune system with plant lectins to combat microbial infections. Front Pharmacol.

[CR20] Kamiya Y, Satoh T, Kato K (2012). Molecular and structural analysis for N-glycan-dependent determination of glycoprotein fates in cells. Biochem Biophys Acta.

[CR21] Klafke GB, Borsuk S, Gonçales SA, Arruda FVS, Carneiro VA, Teixeira EH, Coelho da Silva AL, Cavada BS, Dellagostin AO, Pinto LS (2013). Inhibition of initial adhesion of oral bacteria through a Lectin from *Bauhinia variegata* L. var variegata expressed in *Escherichia coli*. J Appl Microbiol.

[CR22] Laemmli UK (1970). Cleavage of structural proteins during the assembly of the head of bacteriophage T4. Nature.

[CR23] Lagarda-Diaz I, Guzman-Partida AM, Vazquez-Moreno L (2017). Legume lectins: proteins with diverse applications. Int J Mol Sci.

[CR24] Lam SK, Ng TB (2009). A protein with antiproliferative, antifungal and HIV-1 reverse transcriptase inhibitory activities from caper *Capparis spinosa* seeds. Phyto med.

[CR25] Lam SK, Ng TB (2011). Lectins: production and practical applications. Appl Microbiol Biotechnol.

[CR26] Moreira RA, Ainouz IL, Oliveira JTA, Cavada BS (1991). Plant lectins, chemical and biological aspects. Mem Inst Oswaldo Cruz.

[CR27] Movafagh A, Ghanati K, Amani D, Mahdavi SM, Hashemi M, Abdolahi DZ, Safari S (2013). The structure biology and application of phytohemagglutinin (pha) in phytomedicine. Archiv Adv Biosci.

[CR28] Nair SS, Madembil NC, Nair P, Raman S, Veerabadrappa SB (2013). Comparative analysis of the antibacterial activity of some phytolectins. J Int Curr Pharm.

[CR29] Oliveira MD, Andrade CA, Santos-Magalhães NS, Coelho LC, Teixeira JA, Carneiro-da-Cunha MG, Correia MT (2008). Purification of a lectin from *Eugenia uniflora* L seeds and its potential antibacterial activity. Lett Appl Microbiol.

[CR30] Patel DK, Kumar R, Laloo D, Hemalatha S (2012). Natural medicines from plant source used for therapy of diabetes mellitus: an overview of its pharmacological aspects. Asia Pac J Tropic Dis.

[CR47] Peumans WJ, Van Damme EJ (1995). Lectins as plant defense proteins. Plant Physiol.

[CR46] Ratanapo S, Ngamjunyaporn W, Chulavatnatol M (2001). Interaction of a mulberry leaf lectin with a phytopathogenic bacterium, P. syringae pv mori. Plant Sci.

[CR31] Rex JH, Pfaller MA, Walsh TJ, Chaturvedi V, Espinel-Ingroff A, Ghannoum MA, Warnock DW (2001). Antifungal susceptibility testing: practical aspects and current challenges. Clin Microbiol Rev.

[CR32] Ryan MP, Rea MC, Hill C, Ross RP (1996). An application in cheddar cheese manufacture for a strain of *Lactococcus lactis* producing a novel broad-spectrum bacteriocin, lacticin 3147. Appl Environ Microbiol.

[CR33] Sharon N, Lis H (1990). Legume lectin a large family of homologous proteins. FASEB J.

[CR34] Sharon N, Lis H (2001). The structural basis for carbohydrate recognition by lectins. Immunol Comp Carbohydr.

[CR35] Silva PM, Lima AL, Silva BV, Coelho LC, Dutra RF, Correia MT (2016). *Cratylia mollis* lectin nanoelectrode for differential diagnostic of prostate cancer and benign prostatic hyperplasia based on label-free detection. Biosens Bioelectron.

[CR36] Sitohy M, Doheim M, Badr H (2007). Isolation and characterization of a lectin with antifungal activity from Egyptian *Pisum sativum* seeds. Food Chem.

[CR37] Sultan NA, Kavitha M, Swamy MJ (2009). Purification and physicochemical characterization of two galactose-specific isolectins from the seeds of *Trichosanthes cordata*. IUBMB Life.

[CR38] Sun J, Yang QL (2011). Purification and identification of a natural lectin from the seed of peanut *Arachis hypogaea*. Open Mater Sci J.

[CR39] Trowbridge IS (1974). Isolation and chemical characterization of a mitogenic lectin from *Pisum sativum*. J Biol Chem.

[CR40] Tsivion Y, Sharon N (1981). Lipid-mediated hemagglutination and its relevance to lectin-mediated agglutination. BBA.

[CR41] Van Damme EJ, Peumans WJ, Pusztai A, Bardocz S (1998). Handbook of plant lectins properties and biomedical applications.

[CR42] Van Damme EJ, Lannoo N, Fouquaert E, Peumans WJ (2003). The identification of inducible cytoplasmic/nuclear carbohydrate-binding proteins urges to develop novel concepts about the role of plant lectins. Glycoconj J.

[CR43] Wang H, Gao J, Ng TB (2000). A new lectin with highly potent antihepatoma and antisarcoma activities from the oyster mushroom *Pleurotus ostreatus*. Biochem Biophys Res Commun.

[CR44] Wong JH, Chan HE, Ng TB (2008). A mannose/glucose-specific lectin from Chinese evergreen chinkapin (*Castanopsis chinensis*). BBA.

[CR45] Zhang J, Shi J, Ilic S, Jun Xue S, Kakuda Y (2008). Biological properties and characterization of lectin from red kidney bean (*Phaseolus vulgaris*). Food Rev Intern.

